# Association of Early and Late Contrast-Associated Acute Kidney Injury and Long-Term Mortality in Patients Undergoing Coronary Angiography

**DOI:** 10.1155/2021/6641887

**Published:** 2021-03-08

**Authors:** Zhubin Lun, Jin Liu, Liwei Liu, Jingjing Liang, Guanzhong Chen, Shiqun Chen, Bo Wang, Qiang Li, Haozhang Huang, Zhidong Huang, Danyuan Xu, Yunzhao Hu, Ning Tan, Jiyan Chen, Yong Liu, Jianfeng Ye

**Affiliations:** ^1^Department of Cardiology, Dongguan TCM Hospital, Dongguan 523000, China; ^2^The First School of Clinical Medicine, Guangdong Medical University, Zhanjiang 523808, China; ^3^Department of Cardiology, Guangdong Provincial Key Laboratory of Coronary Heart Disease Prevention, Guangdong Cardiovascular Institute, Guangdong Provincial People's Hospital, Guangdong Academy of Medical Sciences, Guangzhou 510080, China; ^4^Department of Cardiology, Shunde Hospital of Southern Medical University, Shunde, Guangzhou, China

## Abstract

**Background:**

Contrast-associated acute kidney injury (CA-AKI) is a common complication in patients undergoing coronary angiography (CAG). However, few studies demonstrate the association between the prognosis and developed CA-AKI in the different periods after the operation.

**Methods:**

We retrospectively enrolled 3206 patients with preoperative serum creatinine (Scr) and at least twice SCr measurement after CAG. CA-AKI was defined as an increase ≥50% or ≥0.3 mg/dL from baseline in the 72 hours after the procedure. Early CA-AKI was defined as having the first increase in SCr within the early phase (<24 hours), and late CA-AKI was defined as an increase in SCr that occurred for the first time in the late phase (24–72 hours). The first endpoint of this study was long-term all-cause mortality. Kaplan–Meier analysis was used to count the cumulative mortality, and the log-rank test was used to assess differences between curves. Univariate and multivariate cox regression analyses were performed to assess whether patients who developed different type CA-AKI were at increased risk of long-term mortality.

**Results:**

The number of deaths in the 3 groups was 407 for normal (12.7%), 106 for early CA-AKI (32.7%) and 57 for late CA-AKI (17.7%), during a median follow-up period of 3.95 years. After adjusting for important clinical variables, early CA-AKI (HR = 1.33, 95% CI: 1.02–1.74, *P*=0.038) was significantly associated with mortality, while late CA-AKI (HR = 0.92, 95% CI: 0.65–1.31, *P*=0.633) was not. The same results were found in patients with coronary artery disease, chronic kidney disease, diabetes mellitus, and percutaneous coronary intervention.

**Conclusions:**

Early increases in Scr, i.e., early CA-AKI, have better predictive value for long-term mortality. Therefore, in clinical practice, physicians should pay more attention to patients with early renal injury related to long-term prognosis and give active treatment.

## 1. Introduction

Contrast-associated acute kidney injury (CA-AKI) is a common complication in patients undergoing coronary angiography (CAG), and CA-AKI is associated with prolonged hospitalization, long-term morbidity, and mortality [1–5].

The majority of studies on the long-term outcome of patients after angiography reported that CA-AKI was an independent predictor of mortality [6, 7]. Notably, the predictive power of CA-AKI on outcome is related to different definitions, which commonly refer to the increase in the serum creatinine (SCr) level from baseline to 48–72 hours after contrast exposure [8]. In previous research, CA-AKI (defined as an increase of Scr ≥0.3 mg/dL or ≥50% from baseline) had higher population-attributable risks (PAR) for long-term mortality than other definitions of CA-AKI [9].

Recent evidence suggests that creatinine levels at different postoperative timepoints are related to adverse clinical outcomes [10–12]. Yong et al. observed that increased creatinine levels at 48–72 hours postsurgery have similar good predictive value for long-term mortality as early increases in creatine [13]. Ribichini et al. found that the increase of SCr in the early phase (12 hours) had better diagnostic power than other phases (24 and 48 hours) for predicting 30-day renal damage after exposure to contrast media [14, 15]. However, in these studies, the association of the changes in postoperative creatinine levels in patients who developed CA-AKI with long-term mortality was limited, which reduced the ability to assess the predictive value of CA-AKI compared with different definitions. In addition, the definitions that are strongest predictors of mortality and their relationship with long-term outcome in the different time courses remain uncertain. Meanwhile, evidence for the influence of CA-AKI on prognosis is derived mainly from the ST-elevated myocardial infarction and acute myocardial infarction populations.

Therefore, it is desirable to evaluate the association between the occurrence of CA-AKI in different time periods and long-term mortality among patients undergoing CAG.

## 2. Methods

### 2.1. Enrollment and Treatment

**Figure 1:**
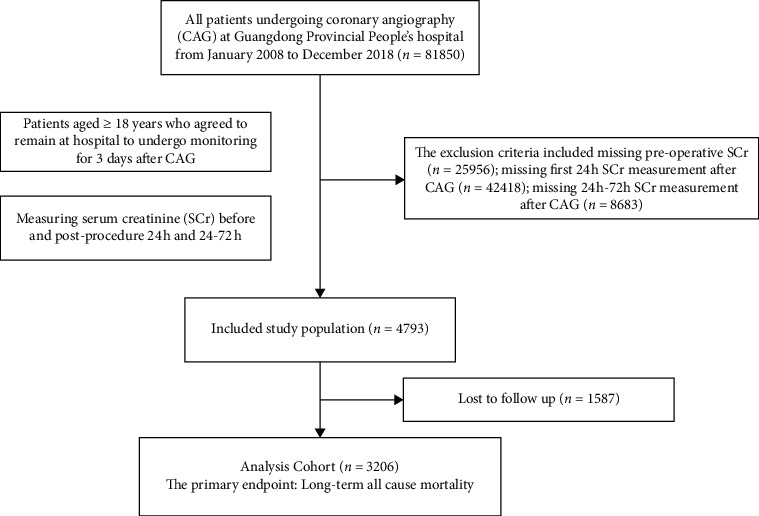
Study flow chart.

This observational study included all consecutive patients who underwent CAG at Guangdong Provincial People's Hospital between January 2008 and December 2018 (*n* = 81,850). The patients aged  ≥ 18 years who agreed to remain at hospital to undergo monitoring for 3 days after CAG were included in the study. Exclusion criteria included missing preoperative SCr values, missing first 24 h SCr values, and 24–72 h SCr values after CAG. Patients who lack follow-up data were also excluded. There were in total 81,850 potentially eligible patients, of whom 3,206 had preoperative and postoperative Scr values and therefore could be included for analysis (Figure 1). The study was approved by the Ethics Committee of Guangdong Provincial People's Hospital.

In accordance with standard clinical practice, CAG was performed through the femoral or radial approach using standard guide catheters, guidewires, balloon catheters, and stents. The treatment of patients was based on guidelines from the American Heart Association/American College of Cardiology Foundation [16].

### 2.2. Endpoint and Definitions

The endpoint of this study was long-term all-cause mortality. All eligible patients included were retrospectively followed up through office visits or telephone interviews. CA-AKI defined as an increase ≥50% or ≥0.3 mg/dL from baseline in the 72 hours after the procedure [17]. Early CA-AKI was defined as having the first increase in SCr within the early phase (<24 hours), and late CA-AKI was defined as an increase in SCr that occurred for the first time in the late phase (24–72 hours). The normal kidney function was defined as normal kidney function or patients with increase <50% or <0.3 mg/dL in creatinine in the 72 hours after angiography. The estimated glomerular filtration rate (eGFR) was calculated by applying the Modification of Diet in Renal Disease (MDRD) equation. Chronic kidney disease (CKD) was defined as an eGFR < 60 mL/min/1.73 m^2^. Anemia was defined as a baseline hematocrit value < 39% for men or <36% for women according to the World Health Organization criteria. Congestive heart failure (CHF) was defined as New York Heart Association (NYHA) functional class > 2, Killip class > 1, or pulmonary edema. Diabetes mellitus (DM) was defined as a previous diagnosis of diabetes or an HbAlc level ≥ 6.5 (48 mmol/mol).

### 2.3. Statistical Analysis

According to the endpoints, all patients were divided into three groups (normal, early CA-AKI, and late CA-AKI). Continuous variables were compared using one-way analysis of variance (ANOVA) and presented as the mean ± SD or median ± IQR. The Pearson chi-squared test was used to analyze categorical data, and the categorical data are expressed as counts (percentages). Kaplan–Meier (K-M) analysis was used to determine the cumulative mortality, and a log-rank test was used to assess differences between curves. Univariate and multivariate Cox regression analyses were performed to assess whether patients who developed different types of CA-AKI had an increased risk of long-term death. The data were analyzed based on available cases, and missing data were not included. For multivariate models, cases with missing values for included factors were excluded listwise. Candidate predictors that were significantly different at *P* < 0.05 in univariate analysis for CA-AKI versus no CA-AKI and were clinically relevant were included in the Cox regression models. Subgroup analyses were conducted in patients with coronary artery disease (CAD), CKD, and DM. Sensitivity analysis was performed using an additional definition of CA-AKI (defined as an increase ≥ 25% or ≥ 0.5 mg/dL from baseline in the 72 hours after the procedure). All data analyses were performed using *R* software (version 3.6.5; *R* Foundation for Statistical Computing, Vienna, Austria). A 2-tailed *P* value < 0.05 indicated significance for all analyses.

## 3. Results

### 3.1. Clinical and Procedural Characteristics

A total of 3,206 consecutive patients who underwent CAG were included in the study (Figure 1). The patients were divided into three different groups: 2,530 (78.91%) patients with no CA-AKI, 354 (11.04%) patients with early CA-AKI and 322 (10.04%) patients with late CA-AKI. Additionally, there were 668 (20.85%) patients with acute myocardial infarction (AMI), 498 (15.53%) patients with chronic kidney disease (CKD), 886 (27.61%) patients with diabetes mellitus (DM), and 849 (26.69%) patients with chronic heart failure (CHF). The usage rates of ACEIs/ARBs, beta-blockers, statins, and diuretics were 53.05%, 60.67%, 60.09%, and 42.89%, respectively. Overall, the mean age was 63.75 ± 11.02 years, and men made up 70.84% of the total population (Table 1).

### 3.2. Primary Outcomes

**Figure 2:**
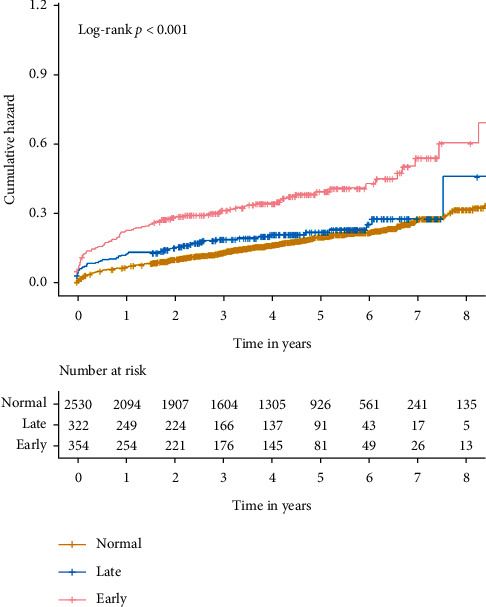
Kaplan–Meier curves for the cumulative probability of mortality stratified according to early CA-AKI, late CA-AKI, and normal. CA-AKI is defined as an increase ≥50% or ≥0.3 mg/dL from baseline.

**Figure 3:**
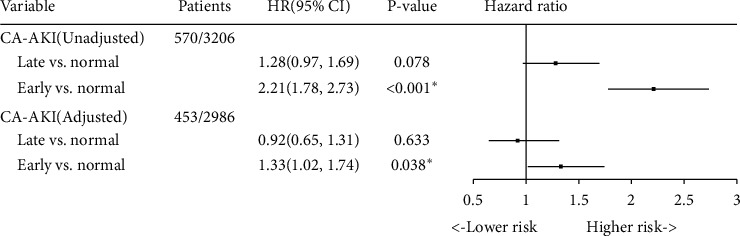
Multivariable analysis for mortality stratified according to early, late, and normal. Adjusted for age≥75, male, acute myocardial infarction, chronic heart failure, hypertension, anemia, contrast media volume, beta-blocker uses, statin use, and diuretic uses; CA-AKI defined as an increase ≥50% or ≥0.3 mg/dL from baseline.

During the follow-up period (median, 3.95 years), 570 patient deaths occurred. The number of deaths in the 3 groups was 407 for normal (12.7%), 106 for early CA-AKI (32.7%), and 57 for late CA-AKI (17.7%). Kaplan–Meier curve analysis revealed that there are significant statistical differences between normal, early CA-AKI, and late CA-AKI (*P* < 0.001, Figure 2). In the Cox proportional hazards model, after adjustment for age ≥ 75, male, acute myocardial infarction, chronic heart failure, hypertension, anemia, contrast media volume, beta-blocker uses, statin use, and diuretic uses, early CA-AKI was significantly associated with mortality (HR: 1.33, 95% CI:1.02–1.74, *P*=0.038), while late CA-AKI was not (HR: 0.92, 95% CI:0.65–1.31, *P*=0.633) (Table 2 and Figure 3).

Our results showed that early CA-AKI and late CA-AKI were significantly associated with in-hospital death after adjusting age, chronic heart failure, and acute myocardial infarction (OR: 9.95, 95% CI: 4.70–21.04, *P* < 0.001 and OR: 4.75, 95% CI: 1.84–12.28, *P*=0.001, respectively) (Supplement Table 1).

### 3.3. Sensitivity Analysis

**Figure 4:**
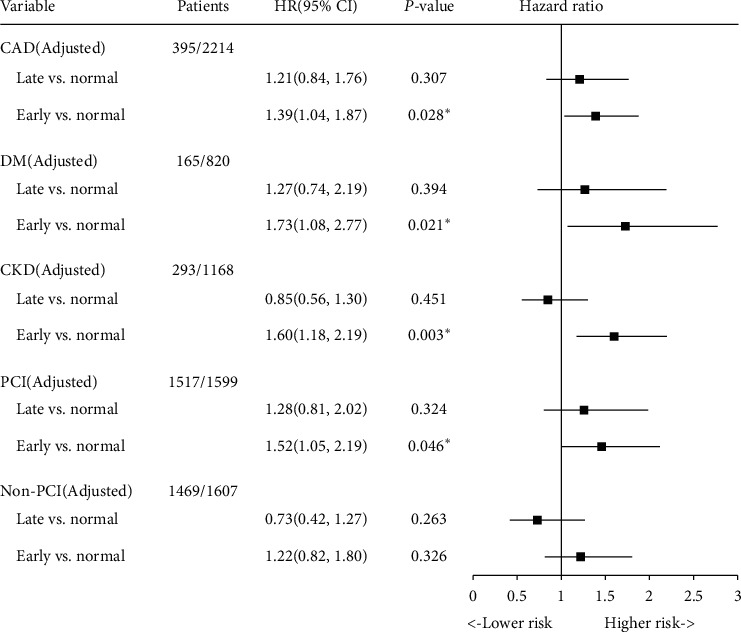
Multivariable analysis for mortality stratified according to early CA-AKI, late CA-AKI, and normal in patients with CAD, CKD, DM, PCI, and Non-PCI. CA-AKI defined as an increase ≥50% or ≥0.3 mg/dL from baseline. Adjusted for age≥75, male, acute myocardial infarction, chronic heart failure, hypertension, anemia, contrast media volume, beta-blocker uses, statin use, and diuretic uses.

We conducted the same analysis in patients with CAD and found that early CA-AKI was significantly correlated with long-term mortality (HR: 1.39, 95% CI: 1.04–1.87, *P*=0.028), while late CA-AKI was not significantly correlated (HR: 1.21, 95% CI: 0.84–1.76, *P*=0.307). At the same time, we found similar results in patients with DM, CKD, or PCI. However, we found that early and late CA-AKI were not associated with long-term mortality in non-PCI patients (HR: 1.30, 95% CI: 0.89–1.92, *P*=0.179 and HR: 4.75, 95%CI: 1.84–12.28, *P*=0.001, respectively) (Figure 4).

**Figure 5:**
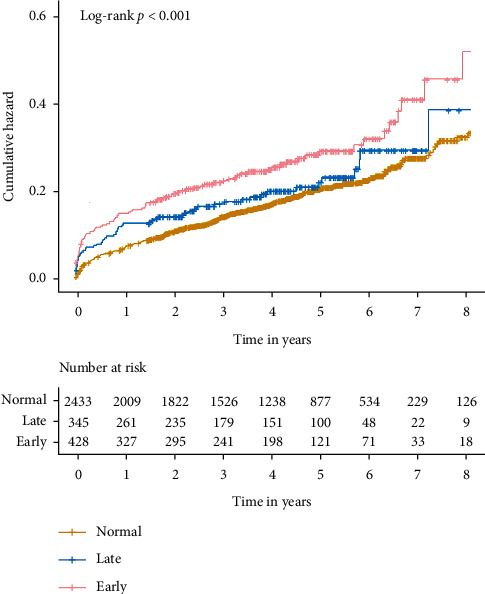
Kaplan–Meier curves for the cumulative probability of mortality stratified according to early CA-AKI, late CA-AKI, and normal. CA-AKI is defined as an increase ≥25 or ≥0.5 mg/dL from baseline.

When using different criteria, e.g., CA-AKI_0525_ (an increase ≥ 25% or ≥ 0.5 mg/dL from baseline in the 72 hours after the procedure), Kaplan–Meier curve analysis revealed that there are significant statistical differences between normal, early CA-AKI, and late CA-AKI (*P* < 0.001, Figure 5).

## 4. Discussion

We compared the relationship between the onset of CA-AKI and long-term mortality in patients undergoing CAG. According to our analysis, patients with early CA-AKI had a worse prognosis than patients without CA-AKI. Moreover, we also found similar results in CA-AKI which defined as a serum creatine elevation ≥25% or ≥0.5 mg/dl within 72 hours. Precise interventions for high-risk groups are of great significance for reducing mortality, medical resource use, and medical costs in clinical practice.

In our cohort, the incidence of CA-AKI was 21.09%, which was higher than the incidence reported by George Chalikias [18]. At a median follow-up of 3.95 years, the mortality was 17.78%, which was slightly lower than the mortality reported by Sinkovic et al. [19]. Sinkovič included patients with STEMI in the study, which may have led to increased mortality. When using the definition of CA-AKI as a serum creatine elevation ≥50% or ≥0.3 mg/dl within 72 hours, we unexpectedly found that early CA-AKI can increase the risk of long-term mortality, while the long-term prognosis of late CA-AKI was similar to that of patients without CA-AKI. This means that patients with CA-AKI within 24 hours after surgery have a poor long-term prognosis. In previous studies, early CA-AKI was shown to increase the risk for long-term mortality, and the results showed that late CA-AKI was significantly related to long-term mortality, which contradicts our conclusions [13]. The possible reason is that the median follow-up time of Liu et al.'s study was 2.45 years, while the median follow-up time of our study was 3.95 years. After prolonging the follow-up time, patients with late CA-AKI no longer had an increased mortality rate.

After exposure to contrast media, kidney damage can occur due to early hemodynamic instability, and 4–6 hours after the procedure is when contrast media excretion occurs [5, 20, 21]. Contrast media damages renal tubular epithelial cells, leading to loss of function, apoptosis, and necrosis [5]. SCr is a clinically significant and sensitive indicator of renal function, and an increase in SCr is associated with poor long-term and short-term outcomes [22]. Ribichini et al. found that the early increase of SCr (12 hours from baseline) had better diagnostic power for predicting short-term kidney damage, but they did not discuss the relationship with long-term prognosis [15]. At present, the new diagnosis and treatment technology allows for patients to be discharged within 24 hours after CAG, which may affect the assessment of the poor prognosis of CA-AKI. Therefore, patients with early CA-AKI should receive additional renal treatment. The long-term prognosis of late CA-AKI patients is similar to that of patients without CA-AKI. This indicates that CA-AKI on the second and third days after surgery will not affect the prognosis and should not interfere with the current treatment plan.

In the subgroup analysis, when we adjusted for some important clinical variables (such as CKD, DM, and CAD), we also found that only early CA-AKI was associated with long-term prognosis. When we adjusted for multiple baseline variables, the results remain unchanged, which indicates that our results are consistent. Late CA-AKI is more likely to be related to creatinine fluctuations. When using the definition of CA-AKI as a serum creatine elevation ≥25% or ≥0.5 mg/dl within 72 hours, we found similar results, which further confirmed our conclusion. The clinical and research significance of this study is that we should focus on the SCr level on the first day after CAG surgery to identify the high-risk population.

Our research had several limitations. First, our study was a single-center retrospective study, which may have caused our results to be unrepresentative. However, the follow-up period of our study was 6 years, which made our research results more credible. Second, our outcome event was only long-term follow-up mortality, and there was a lack of endpoints including in-hospital death, rehospitalization, and assessment of renal function during follow-up. These outcomes are unfavorable for short-term assessment of patients' health-related economic burden and long-term assessment of the impact on all-cause mortality. Third, we defined early CA-AKI and late CA-AKI based on previous research, which may have affected our conclusions. However, after adjusting for important clinical variables, we found similar results in other populations (CAD, CKD, and DM), which indicates that our results are credible. Fourth, adequate periprocedural IV saline hydration for the prevention of CA-AKI should was recommended in previous guidelines [23]. Since we are unable to obtain the patient's hydration data from the hospital system, we cannot adjust the hydration in the multivariable analysis. But, we have adjusted some important risk factors at the same time, which will make our results more credible.

## 5. Conclusion

Our results confirm that early CA-AKI patients are significantly associated with long-term mortality after CAG, while late CA-AKI patients are not significantly associated with long-term mortality. This means that in clinical practice, physicians should pay more attention to patients with early CA-AKI and provide active treatment.

## Figures and Tables

**Table 1:** Baseline characteristics.

Characteristic	Overall (*n* = 3,206)	Normal (*n* = 2,530)	Late **CA-AK** (*n* = 322)	Early **CA-AKI** (*n* = 354)	*P* value
Age, years	63.75 (11.02)	63.61 (10.97)	63.52 (10.54)	64.89 (11.73)	0.1148
Age > 75, years (%)	584 (18.22)	441 (17.43)	57 (17.70)	86 (24.29)	0.0071
Men, *n* (%)	2271 (70.84)	1828 (72.25)	191 (59.32)	252 (71.19)	<0.001
Diabetes mellitus, *n* (%)	886 (27.64)	717 (28.34)	76 (23.60)	93 (26.27)	0.1673
AMI, *n* (%)	668 (20.85)	542 (21.44)	45 (13.98)	81 (22.88)	0.023
Hypertension, *n* (%)	1744 (54.40)	1408 (55.65)	132 (40.99)	204 (57.63)	<0.001
CKD, *n* (%)	498 (15.53)	342 (16.85)	67 (8.15)	89 (25.14)	<0.001
CHF, *n* (%)	849 (26.69)	616 (24.49)	111 (34.91)	122 (35.06)	<0.0001
CAD, *n* (%)	2371 (73.98)	1968 (77.82)	163 (50.62)	240 (67.80)	<0.001
PCI, *n* (%)	1599 (49.88)	1365 (53.95)	97 (30.12)	137 (38.70)	<0.001
Vascular disease, *n* (%)	713 (22.25)	595 (23.54)	54 (16.77)	64 (18.08)	<0.001
Anemia, *n* (%)	161 (5.06)	115 (4.61)	19 (5.94)	31 (8.81)	<0.001
CMV, ml	163.94 (112.43)	169.90 (112.32)	119.10 (98.03)	162.15 (116.40)	<0.001
LDLC, mmol/L	2.79 (0.96)	2.81 (0.99)	2.72 (0.85)	2.78 (0.88)	0.3550
HDLC, mmol/L	0.99 (0.28)	0.99 (0.28)	1.03 (0.31)	0.99 (0.28)	0.0485
Cys-C, mg/L	1.43 (0.81)	1.36 (0.74)	1.69 (0.91)	1.95 (1.18)	<0.0001
eGFR, ml/min/1.73 m^2^	68.74 (28.58)	69.59 (27.83)	66.82 (29.83)	64.38 (32.07)	0.0025
Creatine kinase, U/L	261.66 (598.67)	92.00 (59.05, 164.50)	82.00 (54.00, 191.25)	96.00 (59.00, 176.30)	0.2441
NT-proBNP, pg/mL	3724.79 (7141.08)	877.50 (187.15, 3045.00)	1359.00 (527.80, 4260.00)	1684.00 (536.80, 5173.00)	<0.001
		877.50 (187.15, 3045.00)	1359.00 (527.80, 4260.00)	1684.00 (536.80, 5173.00)	
NYHA class ＞ 1	1791 (92.22)	1373 (91.29)	216 (94.74)	202 (96.19)	0.0147
Beta-blocker use	1839 (60.67)	1515 (62.17)	149 (51.20)	175 (57.76)	<0.001
Statin use	2094 (69.09)	1817 (74.56)	108 (37.11)	169 (55.78)	<0.001
Diuretic use	1300 (42.89)	893 (36.64)	219 (75.26)	188 (62.05)	<0.001
ACEI/ARB use	1608 (53.05)	1400 (57.45)	93 (31.96)	115 (37.95)	<0.001

Abbreviations: AMI, acute myocardial infarction; CKD, chronic kidney disease; CHF, chronic heart failure; CAD, coronary artery disease; HDL-C, high-density lipoprotein cholesterol; LDL-C, low-density lipoprotein cholesterol; LVEF, left ventricular ejection fraction; eGFR, estimated glomerular filtration rate; BUN, blood urea nitrogen; PCI, percutaneous coronary intervention; CMV, contrast media volume; ACEI/ARB, angiotensin-converting enzyme inhibitor/angiotensin receptor blocker.

**Table 2:** Univariable and multivariable analysis of risk factors for long-term mortality.

	Univariable analysis			Multivariable analysis		
	HR	95% CI	*P* value	HR	95% CI	*P* value
Age ≥ 75	2.52	2.12–3.00	<0.001	2.28	1.86–2.80	<0.001
Male	1.28	1.06–1.55	0.011	1.39	1.12–1.73	0.003
CHF	2.46	2.08–2.91	<0.001	1.84	1.49–2.28	<0.001
AMI	1.55	1.29–1.87	<0.001	0.90	0.71–1.13	0.359
Anemia	2.64	2.03–3.45	<0.001	1.91	1.40–2.60	<0.001
Hypertension	1.57	1.32–1.87	<0.001	1.52	1.23–1.88	<0.001
Beta-blocker	1.04	0.86–1.25	0.697	1.01	0.83–1.23	0.900
Diuretic	1.76	1.47–2.12	<0.001	1.75	1.42–2.15	<0.001
Statin	1.52	1.21–1.92	<0.001	1.37	1.05–1.79	0.020
CMV	1.00	1.00–1.00	0.514	1.00	1.00–1.00	0.769
CA-AKI						
Normal	Ref	Ref	Ref	Ref	Ref	Ref
Late	2.21	0.97–1.69	0.078	0.92	0.65–1.31	0.633
Early	1.28	1.78–2.73	<0.001	1.33	1.02–1.31	0.038

Abbreviations: AMI, acute myocardial infarction; CHF, chronic heart failure; CMV, contrast media volume; CA-AKI, contrast-associated acute kidney injury.

## Data Availability

The datasets used and/or analyzed during this study are available from the corresponding author on reasonable request.

## Supplementary Materials

Supplementary Materials Supplement Table 1: univariable and multivariable analyses of risk factors for in-hospital death.
