# Supplementary material for: Association of Early and Late Contrast-Associated Acute Kidney Injury and Long-Term Mortality in Patients Undergoing Coronary Angiography
Source: J Interv Cardiol. 2021 Mar 8;2021:6641887. doi: 10.1155/2021/6641887 (PMC8074549; doi:10.1155/2021/6641887)
Supplement: Supplementary Materials — Supplement Table 1: univariable and multivariable analyses of risk factors for in-hospital death. [file 6641887.f1.doc]

**Supplement Table 1. Univariable and multivariable analysis of risk factors for in-hospital death.**

|  | **Univariable analysis** | | | **Multivariable analysis** | | | |
| --- | --- | --- | --- | --- | --- | --- | --- |
|  | OR | 95% CI | P-value | | OR | 95% CI | P-value |
| Age≥75 | 3.05 | 1.61-5.78 | ＜0.001 | | 2.37 | 1.19-4.72 | 0.014 |
| Male | 1.66 | 0.76-3.61 | 0.204 | |  |  |  |
| CHF | 2.36 | 1.23-4.53 | 0.010 | | 1.51 | 0.75-3.02 | 0.248 |
| AMI | 2.57 | 1.36-4.86 | 0.004 | | 2.37 | 1.19-4.72 | 0.043 |
| Anemia | 2.11 | 0.74-6.00 | 0.161 | |  |  |  |
| Hypertension | 1.75 | 0.90-3.41 | 0.098 | |  |  |  |
| CMV | 1.00 | 1.00-1.00 | 0.327 | |  |  |  |
| CA-AKI |  |  |  | |  |  |  |
| Normal | Ref | Ref | Ref | | Ref | Ref | Ref |
| Late | 6.03 | 2.52-14.44 | ＜0.001 | | 4.75 | 1.84-12.28 | 0.001 |
| Early | 11.90 | 5.73-24.74 | ＜0.001 | | 9.95 | 4.70-21.04 | ＜0.001 |

Abbreviations: AMI, acute myocardial infarction; CHF, chronic heart failure; CA-AKI, contrast-associated acute kidney injury; OR, Odds Ratio;
